# Two sampling methods yield distinct microbial signatures in the nasopharynges of asthmatic children

**DOI:** 10.1186/s40168-016-0170-5

**Published:** 2016-06-16

**Authors:** Marcos Pérez-Losada, Keith A. Crandall, Robert J. Freishtat

**Affiliations:** Computational Biology Institute, George Washington University, Innovation Hall, Suite 305, 45085 University Drive, Ashburn, VA 20147 USA; Division of Emergency Medicine, Children’s National Medical Center, Washington, DC 20010 USA; CIBIO-InBIO, Centro de Investigação em Biodiversidade e Recursos Genéticos, Universidade do Porto, Campus Agrário de Vairão, Vairão, 4485-661 Portugal

**Keywords:** 16S rRNA, Asthma, Microbiome, Microenvironment, Nasopharynx

## Abstract

**Background:**

The nasopharynx is a reservoir for pathogens associated with respiratory illnesses, such as asthma. Next-generation sequencing (NGS) has been used to characterize the nasopharyngeal microbiome during health and disease. Most studies so far have surveyed the nasopharynx as a whole; however, less is known about spatial variation (biogeography) in nasal microenvironments and how sampling techniques may capture that microbial diversity.

**Findings:**

We used targeted 16S rRNA MiSeq sequencing and two different sampling strategies [nasal washes (NW) and nasal brushes (NB)] to characterize the nasopharyngeal microbiota in 30 asthmatic children. Nasal brushing is more abrasive than nasal washing and targeted the inner portion of the inferior turbinate. This region is expected to be different from other nasal microenvironments. Nasal washing is not spatially specific. Our 30 × 2 nasal microbiomes generated 1,474,497 sequences, from which we identified an average of 157 and 186 OTUs per sample in the NW and NB groups, respectively. Microbiotas from NB showed significantly higher alpha-diversity than microbiotas from NW. Similarly, both nasal microbiotas were distinct from each other (PCoA) and significantly differed in their community composition and abundance in at least 9 genera (effective size ≥1 %).

**Conclusions:**

Nasopharyngeal microenvironments in asthmatic children contain microbiotas with different diversity and structure. Nasal washes and brushes capture that diversity differently. Future microbial studies of the nasopharynx need to be aware of potential spatial variation (biogeography).

## Findings

### Background

The nasopharynx is considered an anatomical reservoir from which pathogenic microbes can spread to the lower and upper respiratory airways and cause respiratory infections [1–3]. Culture-independent sequencing methods have shown that some pathogenic bacterial genera associated with asthma (e.g., *Moraxella*, *Streptococcus*, *Haemophilus*, *Neisseria*, and *Staphylococcus*) are also present in the nasopharynx [1, 4–6]. Consequently, several recent metataxonomic and metagenomic (see [7] for distinction) studies have investigated how nasopharyngeal microbial communities change during health and disease in relation to clinical variation [1, 3, 5, 8–16]. All these next-generation sequencing (NGS) studies, however, either sampled the nasopharynx as a whole or focused on a particular anatomical area (microenvironment); hence, less is known about the spatial variation (biogeography) in microbial composition of the nasopharynx. The nose has a complicated and diverse anatomical structure, comprised of diverse epithelial cells and glands with different physiologies and functions [17, 18]. Hence, it seems reasonable to expect that different microenvironments in the nose will also harbor distinct microbial communities. However, to our knowledge, only one study has assessed the biogeography of the nasal microbiota [19]. In that study, the authors used 16S rRNA sequence data to compare the microbiotas of three nasal sites (anterior naris, middle meatus, and sphenoethmoidal recess) in healthy subjects and detected significant differences in diversity between the anterior nares and the two inner mucosal sites. No study, so far, has investigated the biogeography of the nasal cavity in asthmatic patients.

In this report, we used targeted 16S rRNA sequencing and two different sampling techniques (nasal washes and nasal brushes) to characterize the nasopharyngeal microbiota in asthmatic children. Nasal brushing is more abrasive than nasal washing and was used to target a particular region of the nasopharynx, the inner portion of the inferior turbinate; nasal washing is assumed to be less spatially specific and to reach the main cavities in the nasopharynx. Hence, given these differences in sampling methodology, we hypothesize that nasal microbiotas collected by nasal washes will be different in alpha- and beta-diversity from those collected by nasal brushes.

### Methods

#### Ethics approval and consent to participate

All participants in this study were part of the AsthMaP2 (Asthma Severity Modifying Polymorphisms) Study [20]. AsthMaP2 is an ongoing study of urban children and adolescents designed to find associations among airway microbes, environmental exposures, allergic sensitivities, genetics, and asthma. AsthMaP2 and the study presented here were approved by the Children’s National Medical Center Institutional Review Board (Children’s National IRB), which requires that consent is obtained and documented prior to conducting study procedures and collection of samples for research. Written consent was obtained from all independent participants or their legal guardians using the Children’s National IRB approved informed consent documents (IRB No PRO00002517).

#### Samples and molecular analyses

A total of 30 children and adolescents (ages 6 to 17 years) were recruited from the metropolitan Washington, DC, area. All had been physician-diagnosed with asthma for at least one year prior to recruitment. Individuals who reported a medical history of chronic or complex cardiorespiratory disease were ineligible. Their nasopharynges were simultaneously sampled using both nasal washes (NW) and nasal brushes (NB). NW samples were procured by instilling 5 ml of isotonic sterile saline buffer into each nare, holding it for 10 s, and then blowing into a specimen collection container. NB samples were procured by brushing the mucosa of the inner section of the inferior turbinate of each nostril with a sterile nylon bristle cytology brush (CytoSoft Cytology Brush No. CYB-1, Medical Packaging Corporation Camarillo, CA). That mucosa is covered with a mucus blanket that traps smaller particulate matter and bacteria [21]. Moreover, cytology brushes are designed to harvest epithelial cells, meaning NB sampling is generally more abrasive than NW sampling, and so, more likely to collect the microorganisms closely attached to the nasal mucosa. Nonetheless, since the cytology brushes are not protected, we cannot ensure that these samples represent the inferior turbinate microbial community alone, and it is possible that upon removal of the brush from the sinuses, unintended mucus, hair, and epithelial cells from the nares were also collected. Nasal washes are non-specific and may target a different microbial population than nasal brushes, which will pick up microbes attached to the mucosal surface instead of non-attached microbes along the sinonasal cavity (including the inferior turbinate).

Total DNA was extracted using the QIAGEN QIAamp DNA Kit (Catalog # 51304). Before adding the ATL buffer, samples were pre-incubated in 100 uL of lysozyme-TE buffer pH = 8.0 for 30 min at 37 °C. All extractions yielding >50 ng of total DNA (as indicated by NanoDrop 2000 UV-vis Spectrophotometer measuring) were further processed. DNA extractions were prepared for sequencing using the Schloss’ MiSeq_WetLab_SOP protocol (09.2015) in Kozich et al. [22]. Each DNA sample was amplified for the V4 region (~250 bp) of the 16S rRNA gene, and libraries were sequenced using the Illumina MiSeq sequencing platform at University of Michigan Medical School.

#### Sequence analyses

Raw FASTQ files were processed in mothur v1.35.1 [23]. Default settings were used to minimize sequencing errors as described in Schloss et al. [24]. Clean sequences were aligned to the SILVA_v123 bacterial reference alignment at www.mothur.org. Chimeras were removed using uchime [25], and non-chimeric sequences were classified using the naïve Bayesian classifier of Wang et al. [26]. Sequences were clustered into Operational Taxonomic Units (OTUs) at the 0.03 threshold (species level). OTU sequence representatives and taxonomy were imported (BIOM format) into QIIME [27] for subsequent analyses. The mothur OTU table was filtered to a minimum of two observations (sequences) per OTU. Samples were subsampled (rarefaction analysis) to the smallest sample size (1,100 sequences) to remove the effect of sample size bias on community composition. Nonetheless, OTU differential abundance tests that take advantage of full sample sizes (metagenomeSeq zero-inflated Gaussian [28]) were also applied for comparison.

Trees for phylogenetic diversity calculations were constructed using FastTree [29]. Taxonomic alpha-diversity was estimated as the number of observed OTUs and by the Chao1, Simpson, and Shannon indexes. Phylogenetic alpha-diversity (PD) was calculated by the Faith’s phylogenetic diversity index [30]. Similarly, both taxonomic (Bray-Curtis and Euclidean) and phylogenetic (unweighted and weighted UniFrac) beta-diversity metrics were calculated. Community dissimilarity was estimated using principal coordinates analysis (PCoA). Alpha- and beta-diversity metrics were compared between samples grouped by sampling strategy (NW versus NB) using both parametric and non-parametric versions of the *t* test. Taxonomic and phylogenetic distances were also compared among those groups using the non-parametric PERMANOVA test from the vegan R’s library [31]. Significance was determined through 10,000 permutations. Finally, OTU abundance differences between NW and NB groups and between paired samples (e.g., NW patient 1 versus NB patient 1) were assessed using rarefied (White’s non-parametric *t* test [32] and Fisher’s exact test, respectively) and non-rarefied (metagenomeSeq zero-inflated Gaussian) data. Core microbiome analyses were also performed to identify resident bacteria. Bonferroni or Benjamini-Hochberg FDR multiple test correction methods were applied. All analyses were performed in mothur, QIIME, STAMP [33], and RStudio [34].

#### Availability of the data and materials

Sequence data have been uploaded to the GenBank under SRA accession number SRP069020. All materials used for nasal brushing and washing were processed as regular samples using the same QIAGEN DNA purification kit and PCR protocols. No PCR band was visible on an agarose gel.

### Results

#### Sequences and OTUs

Sixty nasal microbiomes corresponding to 30 asthmatic children were analyzed via MiSeq sequencing of 16S rRNA V4 amplicons. A total of 1,474,497 sequences ranging from 1100 to 62,148 sequences per sample (mean = 24,575; median = 20,599) were obtained after quality control analyses and OTU filtering. From these data, we identified 33–272 OTUs (mean = 157) per sample in the NW group and 120–408 OTUs (mean = 186) per sample in the NB group.

#### Microbial composition of the nasopharynx

**Fig. 1:**
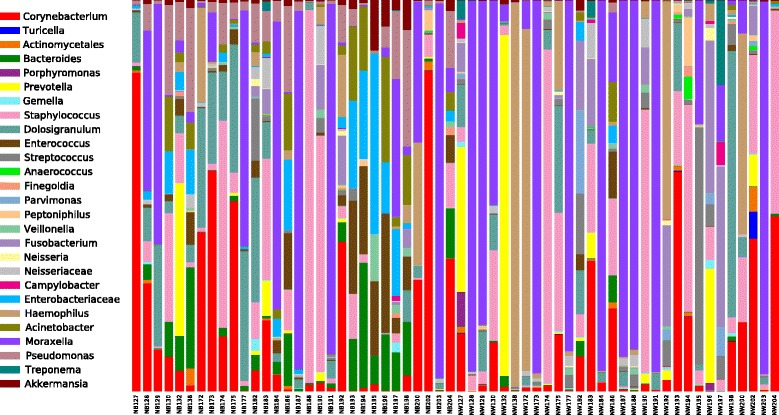
Taxonomic profiles of most abundant bacterial genera (OTU proportion >0.1 %) in 60 nasopharyngeal microbiomes from 30 asthmatic children. *NW* nasal wash, *NB* nasal brush

Nasopharyngeal microbiomes were dominated by the following seven genera, which showed different proportions in NW and NB groups: *Moraxella* (NW = 38.1 %, NB = 27.8 %), *Staphylococcus* (NW = 15.4 %, NB = 10.2 %), *Corynebacterium* (NW = 8.6 %, NB = 20.4 %), *Haemophilus* (NW = 8.4 %, NB = 4.2 %), *Fusobacterium* (NW = 5.8 %, NB = 1.1 %), *Prevotella* (NW = 3.8 %, NB = 1.1 %), and *Dolosigranulum* (NW = 3.7 %, NB = 8.7 %) (see Fig. 1). The core microbiome analysis (OTUs present in 95 % of the samples) identified five OTUs of the genera *Moraxella*, *Staphylococcus*, *Haemophilus*, *Streptococcus*, and *Enterococcus* in NB and four OTUs of the genera *Moraxella*, *Pseudomonas*, *Enterococcus*, and *Bacteroides* in NW. This suggests that NW and NB may contain different resident bacteria. All dominant genera reported here for asthmatic children and adolescents have been detected in other microbiome studies of the nasopharynx in infants with and without respiratory infections [1, 3, 16, 35], although in different proportions. Similarly, microbial profiles observed in our children also looked different from those described in the nasopharynx of adults [19, 36, 37].

#### Nasopharyngeal washes and brushes sampled different microbiotas

**Fig. 2:**
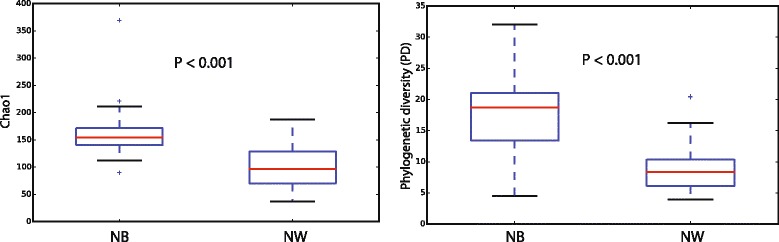
*Box plots* of alpha-diversity indices (Chao1 and phylogenetic diversity) comparing nasal wash (NW) and nasal brush (NB) samples

Microbial profiles of the most abundant bacterial genera (Fig. 1) and alpha-diversity indices (observed OTUs, Chao1, Shannon, Simpson, and PD) varied greatly between NW and NB groups (Fig. 2), with NB showing greater estimates for all indices. These differences were significant in all cases (non-parametric *t* test; *P* < 0.001). A previous NGS 16S metataxonomic study of nasopharyngeal microenvironments showed higher alpha-diversity values (same indices as here) in microbiotas from the posterior sections of the nasopharynx (represented by the middle meatus and sphenoethmoidal recess) than in those from the anterior naris [19]. In accordance with their results, we also see a similar trend in microbiotas mainly collected from the inferior turbinate (NB) compared to those non-specifically sampled along the sinonasal cavity (NW).

**Fig. 3:**
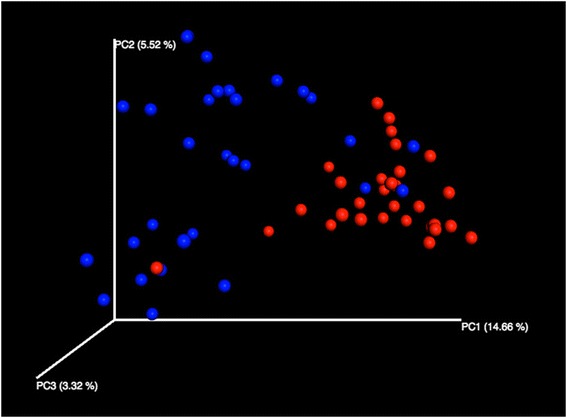
3D principal coordinates analysis (unweighted UniFrac distances) of nasal wash (NW) and nasal brush (NB) samples

**Fig. 4:**
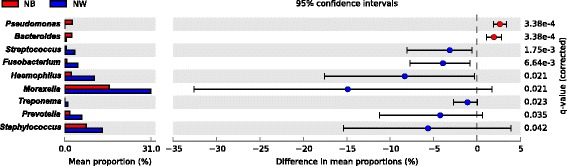
Extended error bar plot showing the nine bacterial genera with a significant difference (White’s test; *P* < 0.05) in proportions of at least 1 % between nasal wash (NW) and nasal brush (NB) samples. Seven genera are overabundant within the nasal microbiotas collected by washing compared to those collected by brushing

Beta-diversity ordination analysis of PCoA showed clear dissimilarities between NW and NB microbiomes for all distances tested (Fig. 3). Similarly, significant differences (*P* < 0.001; *R*^2^ = 0.072) in community composition (PERMANOVA) were observed between both groups after FDR correction. Finally, microbial abundances also significantly varied between NW and NB groups (White’s test; effect size ≥1 % and metagenomeSeq zero-inflated Gaussian) after FDR correction for the following nine genera: *Staphylococcus*, *Prevotella*, *Treponema*, *Streptococcus*, *Moraxella*, *Haemophilus*, *Fusobacterium*, *Bacteroides*, and *Pseudomonas* (Fig. 4). Similarly, all 30 comparisons of paired samples showed significant differences for 3 to 26 OTUs (Fisher’s exact test with an effective size filter of ≥1 %). As above, PCoA analysis in Yan et al. [19] also revealed a separation between anterior nare samples and more posterior mucosal samples. Their ANOVA testing of OTU relative abundances between those two regions detected 18 OTUs that significantly differed between them, although 16 of those relative abundances were <0.1 %. Our analyses showed larger differences in community composition between nasal washes and nasal brushes from potentially different environments in the nasopharynx.

We believe that the observed differences in alpha- and beta-diversity are due to the fact that our brushes tapped on a richer microbial microenvironment in the nasopharynx (inner portion of the inferior turbine) and sampled it more efficiently than the less specific nasal washing. These more diverse microbiotas could represent established bacterial communities in the deeper areas of the nasopharynx, while the less diverse microbiotas sampled by nasal washing would correspond to transient bacterial communities in the nasopharynx.

### Conclusions

With the exception of the vagina, explorations of spatial differences within the human microbiome have been heavily guided by gross anatomical landmarks and boundaries [19]. As a result, microenvironments within otherwise homogenous-appearing human organs, and especially within spatially constrained sites (like the nasopharynx), have been relatively ignored. This report is a first attempt to characterize the nasopharyngeal microbiotas of asthmatic children at a finer spatial scale. It identified distinct microbial signatures in NB and NW samples, which may be due to the distinct microenvironments sampled or the sampling approach itself. Future microbial studies need to be aware of the potential for spatial variation (biogeography) in the nasopharynx and choose the best sampling approach to target the nasal areas of interest. Our next goal is to characterize the nasopharyngeal microbiome in more depth by brushing the nasal vestibule and turbinates (inferior, middle, and superior) to distinguish resident from transient bacteria. Then, we will use this information to compare nasal, oral, and tracheal microbiotas, so airway microbial dynamics during asthma can be better understood.
